# Skin cancers in skin types IV–VI: Does the Fitzpatrick scale give a false sense of security?

**DOI:** 10.1002/ski2.40

**Published:** 2021-06-08

**Authors:** P. Goon, C. Banfield, O. Bello, N. J. Levell

**Affiliations:** ^1^ Department of Dermatology Peterborough City Hospital Edith Cavell Campus Peterborough UK; ^2^ Department of Dermatology Norfolk and Norwich University Hospital Norwich UK

## Abstract

The Fitzpatrick scale has been in use for skin colour typing according to the tanning potential of skin since its inception in 1975–1976. Thomas Fitzpatrick developed the scale to classify persons with ‘white skin’ in order to select the correct amount of UVA in Joules/cm^2^ for PUVA treatment for psoriasis. Since then, it has been widely used in Dermatology to gauge the skin's reaction to UV exposure, tanning potential, assessment of sunburn risk and amount of sun protection required for individual patients. However, the use of this scale has been of limited utility because of different self‐perception in different areas of the world, particularly among those with skin of colour. Skin cancer risk is loosely inversely correlated with the initial genetic/inherent amount of melanin (most research has focused on eumelanin) present in the skin, although the pattern of exposure and amount of UV radiation required causing DNA damage varies widely according to different cancers. In this review, we have shown that the Fitzpatrick scale is neither correct nor adequate to reflect sunburn and tanning risk for skin of colour. Therefore, it may give both patients and physicians a false sense of security that there is little risk that people of colour can develop skin cancers. We have reviewed the small but not insignificant risk of skin of colour developing skin cancers and emphasise that there remains much research that needs to be done in this field.

## Graphical abstract

Fitzpatrick scale skin type IV to VI does not adequately nor accurately define these types, and gives the mistaken impression that skin cancers are not relevant. Skin cancers do occur in these skin types, although at a lower frequency, and more needs to be done in terms of targeting and advising BAME communities for better skin health and sun protection.

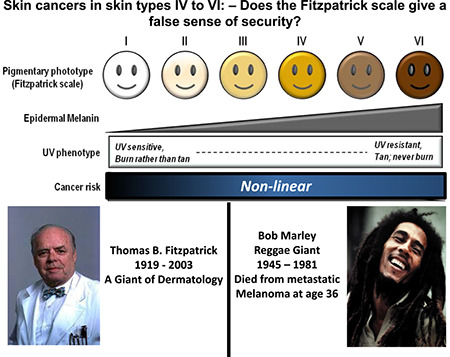

1

**What is already known about this topic?**

- The Fitzpatrick scale was initially developed for typing skin types in order to protect them from UV damage from UVA phototherapy
- Fitzpatrick types VI to VI do not adequately nor accurately portray the wide range of skin tone and types that are normally classified into these 3 categories

**What does this study add?**

- The published evidence demonstrates clearly that skin cancers do occur in skin types VI‐VI, but at a lower level
- BAME communities do not have adequate levels of skin health promotional advice, and sun protection advice
- There is a dearth of dermatology research on skin of colour

**Abbreviations**
Asian: persons tracing their origins to primarily Asian origins
Black: non‐Hispanic persons of primarily African origins
Hispanic: Persons tracing their origins to primarily Central or South American origins
White: non‐Hispanic persons of primarily European Caucasian origins

2

Thomas Fitzpatrick MD (Harvard) developed his eponymously named skin type classification in 1975, as an aid to skin research into the tanning potential of human skin.^1^ As Dr Fitzpatrick himself attested, it was developed to classify persons with white skin in order to select the correct amount of UVA in Joules/cm^2^ for PUVA treatment for psoriasis.^2^ The scale was initially developed with eye and skin colour as the major parameters, but these were misleading, so the ability to tan when exposed to ultra‐violet (UV) radiation was then used as the major parameter.^3^ This scale has largely superseded Von Luschan's chromatic scale, which was widely used in the beginning of the 20th century for anthropometry and racial studies.^4^ This contained 36 different colour variants for racial typing, and has been abandoned by most, in part due to the inaccuracies and inefficiencies inherent in such a user‐dependant system for discriminatory racial profiling (for selective reproduction and sterilisations); see Table 1.

**TABLE 1:** Current Fitzpatrick's type classification based on sun‐tanning potential compared to Von Luschan's numerical classification based on skin colour

Fitzpatrick type	Von Luschan's chromatic scale	Correlation with tanning potential
I	0–6	Always burns, never tans
II	7–13	Usually burns, minimal tanning
III	14–20	Occasionally burns, usually tans uniformly
IV	21–27	Rarely burns, always tans well
V	28–34	Very rarely burns, tans very easily
VI	35–36	Never burns, always tans

Although Fitzpatrick's scale is widely used in Dermatology to help classify the natural pigmentation of human skin and the effects thereon after UV exposure, it is not without its problems. The correlation of skin pigmentation (defined as variable skin tone ranging from very light to very dark) with susceptibility to development of skin cancers is not linear and therefore a six‐category scale is not appropriate for risk assessment as to skin oncogenic potential (likelihood of developing skin cancers) when stated outcomes such as ‘never burns’ or ‘very rarely burns’ are patently not true. Numerous clinical observational studies and epidemiological studies have shown that darker skin has a much reduced risk of developing skin cancer, both melanoma or non‐melanoma skin cancers (NMSC). This is partly due to the photoprotective effect of UV filtering by the increased melanin found in the epidermis. Kaidbey et al.^5^ found that black skin filtered out five times as much UV radiation compared to Caucasian skin so that black skin has been estimated to have an intrinsic SPF of 13.4, in contrast to light skin with an SPF of 3.3. However, it is also very clear that there is no single shade of pigmentation for black skin and there is a wide biological range from light to very dark and any estimates of intrinsic SPF must be taken in this context.

The Fitzpatrick scale has been described as ‘Anglo‐Irish centric’ or ‘simply irrelevant’ when used for skin of colour or outside the Western World. In Europe, Fitzpatrick I–IV may be used to describe the various skin shades common to those with mainly European ancestry, with IV, V and VI also used for brown to black skin, from either central Africa, parts of South America, the South Pacific and Australia and much of the Caribbean including those of mixed ancestry living in other parts of the world. Type V may then be applied to people from many parts of Asia, North Africa and South African, Central and Southern America. However, the scale may be used differently by people from other parts of the world: for example, those in living in parts of Asia or North Africa may consider that the local population has mainly type III or IV skin, and self‐reporting of skin colour has been shown to be influenced by the community that person lives in.^6^, ^7^ Self‐reporting of skin colour using the scale is also known to be less accurate than a trained dermatologist's assessment,^8^ and found to exclude the majority of Black people who self‐report.^9^ The scale is often used wrongly, is not used consistently and therefore is of limited utility.

One of the main problems with the scale is that it reports that type V and VI skin ‘usually or always tans, never or rarely burns’. These categorisations are wholly inaccurate, and reflect attitudes prevalent in the 1960s and 1970s amongst Dermatologists who originated predominantly from Europe or North America. There are studies that show that sunburn amongst darker skin is much more common than previously thought.^10^, ^11^ There has been an epidemic of skin cancer rising in prevalence and incidence amongst the lighter skinned populations in the Western world and almost certainly a slower concomitant rise amongst darker skinned populations as well but this is not reflected in skin cancer registries which are nearly non‐existent in less‐developed countries.

If there has been a rise in skin cancers amongst people of darker skin types, then this should be reflected in skin cancer registries from Asian countries such as Singapore, which have highly developed and modern societies with diverse populations of mainly type III to VI skin types. Indeed, publications such as Sng et al.^12^ document increasing rates of NMSC amongst their population, with the elderly Chinese (type III–IV skin) exhibiting threefold the incidence rates compared to the Malays and Indians who have typically type IV–VI skin. The decreasing rates of skin cancers in skin of increasing pigmentation is confirmed.^13^ We suggest that there is a likely concomitant undocumented rise amongst populations in Africa (particularly Southern Africa where there were waves of migration from Europe and Asia, and consequently a highly mixed population) and the Indian Subcontinent (where the upper castes commonly had lighter coloured skin compared to the lower castes (farmers, labourers, manual workers, etc.).

The commonest skin cancers in people of colour are the NMSCs of which Squamous Cell Carcinomas (SCCs) and Basal Cell Carcinomas (BCCs) comprise the majority. SCCs are the most common skin cancers in people of African and Asian Indian descent, with BCCs next most frequent.^14^, ^15^ Risk factors for NMSC have been studied extensively but nearly all conducted in populations with predominantly less pigmented skin. Cumulative UV exposure is the most important risk factor for NMSC in lighter coloured skin, which also include elderly age, chronic scarring processes, ionising radiation, inflammatory conditions, HPV infection, immunosuppression, genodermatoses such as albinism, xeroderma pigmentosum and so forth.^15^, ^16^, ^17^ Tadokoro et al.^18^ studied melanin content and degree of UVA and B induced DNA damage in normal skin of various ethnic groups. They found that baseline skin eumelanin content was inversely correlated with extent of DNA damage and damage was appreciable in all skin types, even at low levels of exposure. Interestingly, skin of colour was able to more efficiently repair solar‐induced DNA damage than skin from those with light skin. Therefore, the depth of colour of skin (darker skin colour) may be a non‐linear marker indicating more efficient molecular repair processes that help protect skin cell DNA from ionising radiation.

Melanoma incidence was found not to correlate with increased residential UV exposure in skin of colour (Black, Hispanic, Asian and Native Americans).^19^ In contrast, Hu et al.^20^ discovered a positive correlation of melanoma incidence with UV exposure in White, Hispanic and Black people, implying that high intermittent exposure to UV of skin of colour was important in the development of melanoma. The tendency for melanomas and SCCs to develop in UV‐protected areas in skin of colour implies that UV radiation may not be significant for these two skin cancers, in contrast to BCCs (which do develop predominantly in sun‐exposed areas).^21^ These contradictory findings confirm that UV significance in the development of skin cancers in skin of colour remains much under‐researched.

There is solid evidence for a causative role of sunlight (UV radiation) exposure in melanoma oncogenesis, although the relationship between sunlight and melanoma is complex.^22^ Eighty percent of melanomas develop in regions of the world where most light‐skinned people get intermittent intense sun exposure, often during short holidays. Intermittent strong sun exposure and sunburn history, especially in childhood, have been identified as strong risk factors for melanoma.^23^ UV exposure in childhood appears to be a strong driver risk for induction of mutations in the ‘melanocytic system’ and the development of melanocytic naevi. Length of sun exposure, high UV indices and light skin are strongly associated with the development of melanocytic naevi in childhood.^24^, ^25^, ^26^ In adulthood, sunburn and increased sun exposure are associated with the development of solar lentigines and lentigo maligna in sun‐exposed areas.^27^, ^28^

Latitude studies amongst populations with relatively low migration in Europe demonstrate a strong correlation with fatal melanoma incidence in less sunny climates, which in turn, indirectly correlates with skin pigmentation.^22^ By contrast, studies in the US showed that in non‐Hispanic whites, where there has been considerable migration over the last 300 years, there was strong correlation between melanoma incidence and lower latitudes and higher mean UV indices. This effect was also shown in Hispanics and Blacks; thereby strongly suggesting that these risk factors are involved in the same mechanistic pathways towards development of melanoma in all humans.^19^, ^29^ Further epidemiological data demonstrating that intermittent exposure to high doses of UV sufficient to cause sunburn comes from childhood studies on sunburn^23^ and the greatest increase in melanoma incidence appear to be on the torso in men, and legs in women, which are areas of the body exposed to intermittent sun exposure.^30^, ^31^, ^32^

There is much underestimation of the risks of skin cancer in skin of colour, both amongst the general population, and in the communities of people of colour.^11^, ^33^, ^34^ People of colour are also less likely to use sunscreen, and less likely to report sunburn, and have much less familiarity with skin examinations or the need to ask for such examinations. Although the risks of developing skin cancers are lower relative to white populations, these skin cancers tend to be associated with higher morbidity and mortality due to later presentation/delayed diagnoses, and larger tumour volumes.^9^, ^16^, ^21^

In conclusion, we strongly suggest that the Fitzpatrick scale is not appropriate when used for phototyping skin colour in relation to skin cancer risks, especially since tanning and sunburning are not envisaged for people of colour. It has and will continue to give a false sense of security to both physicians and patients. We advocate against its use for people of colour, instead digital photography with standard lighting and the use of artificial intelligence with alternative skin typing systems^35^, ^36^, ^37^ may be better suited to help visualise and objectively categorise skin of colour in relation to UV radiation damage, photoageing and skin cancers. We reiterate that there is a severe lack of data for people of colour developing skin cancers, and that this must be a priority for dermatological research, especially here in the UK, which has an increasingly diverse population in the 21st century.

## CONFLICT OF INTERESTS

The authors declare that there are no conflict of interests.

## Data Availability

Data sharing is not applicable to this article as no new data were created or analyzed in this study
